# *DOK7* Gene Therapy Enhances Neuromuscular Junction Innervation and Motor Function in Aged Mice

**DOI:** 10.1016/j.isci.2020.101385

**Published:** 2020-08-05

**Authors:** Ryo Ueta, Satoshi Sugita, Yoshihiko Minegishi, Akira Shimotoyodome, Noriyasu Ota, Noboru Ogiso, Takahiro Eguchi, Yuji Yamanashi

**Affiliations:** 1 Division of Genetics, Department of Cancer Biology, The Institute of Medical Science, The University of Tokyo, 4-6-1, Shirokanedai, Minato-ku, Tokyo 108-8639, Japan; 2 Biological Science Research, Kao Corporation, 2606 Akabane, Ichikai-machi, Haga-gun, Tochigi 321-3497, Japan; 3 Laboratory of Experimental Animals, The National Center for Geriatrics and Gerontology, 7-430, Morioka-cho, Obu, Aichi 474-8511, Japan

**Keywords:** Clinical Genetics, Molecular Mechanism of Gene Regulation, Musculoskeletal Medicine

## Abstract

Muscle denervation at the neuromuscular junction (NMJ), the essential synapse between motor neuron and skeletal muscle, is associated with age-related motor impairment. Therefore, improving muscle innervation at aged NMJs may be an effective therapeutic strategy for treating the impairment. We previously demonstrated that the muscle protein Dok-7 plays an essential role in NMJ formation, and, indeed, its forced expression in muscle enlarges NMJs. Moreover, therapeutic administration of an adeno-associated virus vector encoding human Dok-7 (*DOK7* gene therapy) suppressed muscle denervation and enhanced motor activity in a mouse model of amyotrophic lateral sclerosis (ALS). Here, we show that *DOK7* gene therapy significantly enhances motor function and muscle strength together with NMJ innervation in aged mice. Furthermore, the treated mice showed greatly increased compound muscle action potential (CMAP) amplitudes compared with the controls, suggesting enhanced neuromuscular transmission. Thus, therapies aimed at enhancing NMJ innervation have potential for treating age-related motor impairment.

## Highlights

• *DOK7* gene therapy enhances motor function and muscle strength in aged (≥2 years) mice
• *DOK7* gene therapy enhances neuromuscular junction (NMJ) innervation in aged mice
• *DOK7* gene therapy increases compound muscle action potential amplitudes in aged mice
• Enhancing NMJ innervation in the elderly may strengthen muscles and motor activities

## Graphical Abstract

**Figure:**
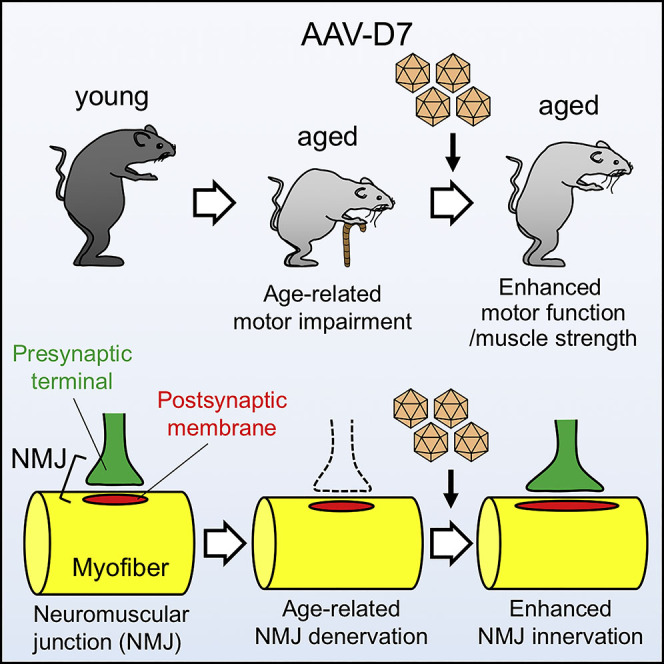

## Introduction

Age-related decline in motor function has a major impact on quality of human life (Hunter et al., 2016). The motor impairment involves age-related changes at least in the nerve and muscle systems, including a pathogenic loss of skeletal muscle mass and strength, known as sarcopenia. Accumulating evidence raises the possibility that the age-related decline in motor function is caused, at least in part, by functional impairment of the neuromuscular junction (NMJ), a cholinergic synapse essential for motoneural control of skeletal muscle contraction (González-Freire et al., 2014; Liu et al., 2017; Punga and Ruegg, 2012; Tintignac et al., 2015). Many studies with rodents have shown age-related denervation at NMJs in addition to degeneration of the presynaptic motor nerve terminals, where the neurotransmitter acetylcholine is released, and the postsynaptic endplate, where acetylcholine receptors (AChRs) densely cluster, suggesting an impaired neuromuscular transmission with aging (Chai et al., 2011; Valdez et al., 2010); however, despite NMJ denervation inevitably leading to the loss of NMJ function, age-related fragmentation of the AChR clusters is not directly associated with the impairment of neuromuscular transmission, indicating that morphological remodeling of the endplate per se is not an accurate predictor of its functional properties (Willadt et al., 2016, 2018). In humans, electrophysiological and muscle fiber-type studies suggested age-related denervation at NMJs (Campbell et al., 1973; Lexell and Downham, 1991; Spendiff et al., 2016). Indeed, it is reported that the denervation rate at NMJs increases upon aging, although age-related morphological changes at NMJs remain controversial (see below; Jones et al., 2017; Oda, 1984; Wokke et al., 1990). Moreover, a recent study suggests that the increased rate of NMJ denervation contributes to the reduction in muscle strength in patients with sarcopenia (Piasecki et al., 2018), supporting the idea that the NMJ is a possible therapeutic target for treating age-related motor dysfunction.

In mammals, the muscle-specific receptor tyrosine kinase MuSK is essential for the formation and maintenance of NMJs (Burden, 2002). The receptor kinase is activated by the motor neuron-derived agrin, which binds to MuSK's coreceptor, low-density lipoprotein receptor-related protein 4 (Lrp4) (Kim et al., 2008; Zhang et al., 2008). Furthermore, activation of MuSK also requires Dok-7 (downstream of tyrosine kinases-7) (Inoue et al., 2009; Okada et al., 2006). Indeed, biallelic mutations in the human *DOK7* gene cause a limb-girdle type of congenital myasthenic syndrome (*DOK7* myasthenia), a disorder characterized by defective NMJ structure or NMJ synaptopathy (Beeson et al., 2006). In addition, we previously generated AAV-D7, a recombinant muscle-tropic adeno-associated virus (AAV) serotype 9 vector carrying the human *DOK7* gene under the control of the cytomegalovirus promoter, and demonstrated that therapeutic administration of AAV-D7—*DOK7* gene therapy—enlarges NMJs, improves the impaired motor activity, and ameliorates the shortened lifespan in mouse models of *DOK7* myasthenia and autosomal dominant Emery-Dreifuss muscular dystrophy, a disease associated with defective NMJs due to genetic mutations in the lamin A/C gene (Arimura et al., 2014; Méjat et al., 2009). Moreover, *DOK7* gene therapy suppressed denervation at NMJs and enhanced motor activity and life span in a mouse model of familial amyotrophic lateral sclerosis (ALS), a fatal neuromuscular disease with motor neuron degeneration (Miyoshi et al., 2017). These findings demonstrate potential for *DOK7* gene therapy in various motor neuron diseases as well as myopathies with NMJ defects. Given that NMJ denervation appears to play a crucial role in age-related decline in motor function similar to that observed in ALS model mice (Valdez et al., 2012), *DOK7* gene therapy might also ameliorate age-related motor impairment by suppressing denervation at NMJs. Thus, in the present study, we examined whether *DOK7* gene therapy improves the motor function in aged mice.

## Results

### *DOK7* Gene Therapy Enhances NMJ Innervation in Aged Mice

Several mouse models with an accelerated aging phenotype have been developed, enabling studies of motor dysfunction and muscle weakness as aging-like phenotypes (Bütikofer et al., 2011; Didier et al., 2012; Hirofuji et al., 2000). However, it remains unclear whether the morphological and functional alterations of NMJs in these mouse models faithfully represent those in aged mice. C57BL/6 mice exhibit morphological NMJ abnormalities and decreased motor function at 24 months of age as compared with those in adulthood (e.g., at 2 or 6 months of age) (Cheng et al., 2013; Graber et al., 2013; Valdez et al., 2010). Because approximately 25% and 90% of C57BL/6 male mice have been reported to die by 24 and 32 months of age, respectively (Turturro et al., 1999), we defined male mice at 24 months of age or older as aged mice in the current study. Indeed, other groups also utilized 24-month-old (“mo” hereafter) mice as aged mice for their studies on age-related alterations of NMJs and motor function (Andonian and Fahim, 1987; Valdez et al., 2010). In addition, it has been reported that motor function declines over time even after 24 months of age (Cheng et al., 2013; Graber et al., 2013).

**Figure 1:**
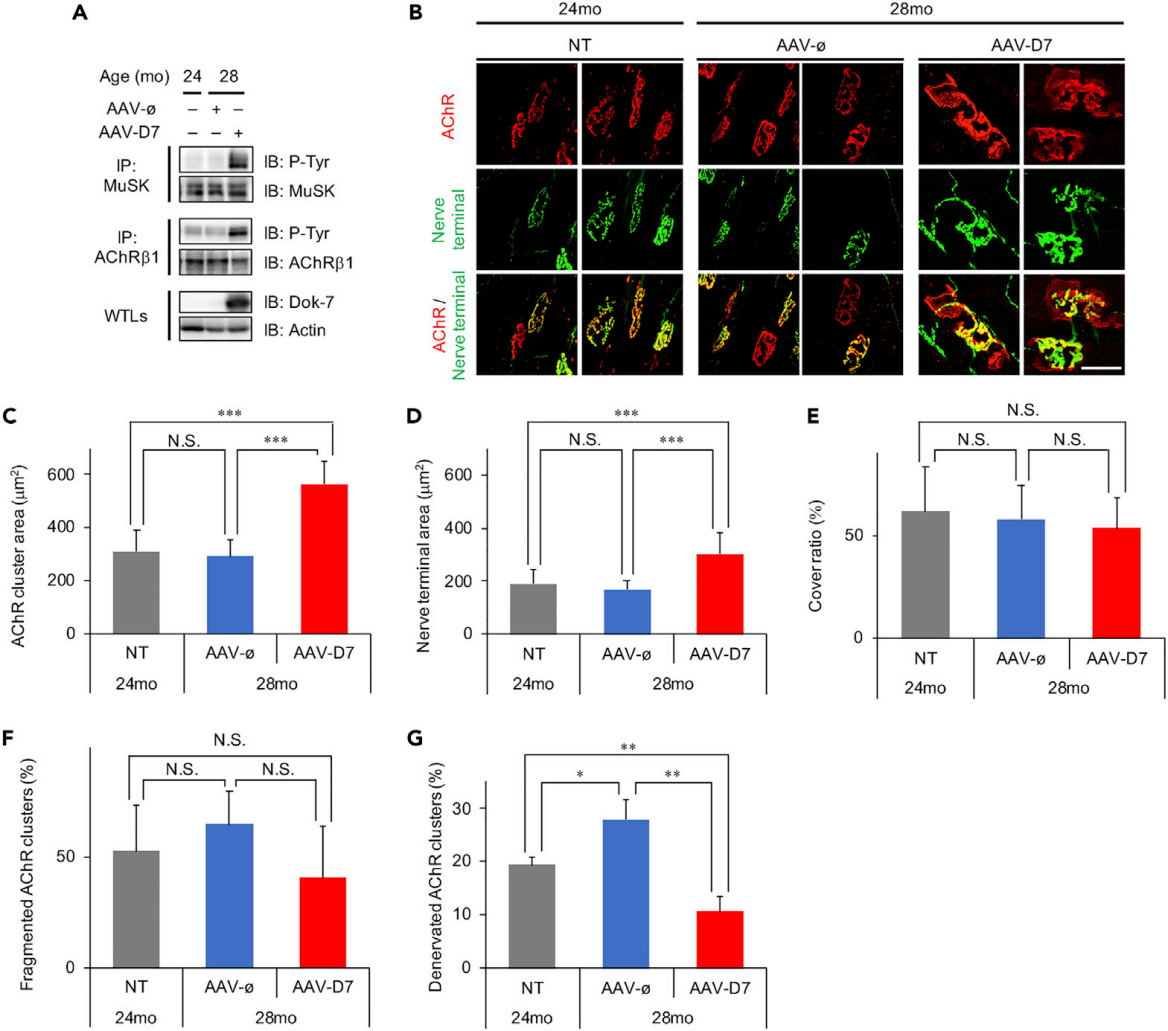
AAV-D7 Treatment Activates MuSK and Enhances NMJ Innervation in Aged Mice Twenty-four month-old (mo) male mice were treated with AAV-D7 or the control empty vector (AAV-ø) and subjected to the following assays at 28 months of age (4 months after the administration of AAV). (A) Anti-MuSK or anti-AChRβ1 immunoprecipitates (IP: MuSK or IP: AChRβ1, respectively) from the hindlimb-muscle lysates of non-treated (−) 24-mo mice, or 28-mo mice treated with AAV-D7 or AAV-ø, together with whole-tissue lysates (WTLs), were subjected to immunoblotting (IB) with the indicated antibodies. P-Tyr, phosphotyrosine. (B–G) Longitudinal sections of tibialis anterior (TA) muscles of non-treated (NT) 24-mo mice or 28-mo mice treated with AAV-D7 or AAV-ø were stained with α-bungarotoxin (AChR) and antibodies to synapsin-1 (Nerve terminal), and representative images are shown (B) (scale bar, 50 μm). The area of AChR clusters (C) and motor nerve terminals (D), the cover ratio (E), and the percentage of fragmented AChR clusters (F), and denervated AChR clusters (G) were quantified. Error bars indicate means ± SEM (*n* = 15 mice for NT 24-mo mice; *n* = 8 mice for AAV-ø-treated 28-mo mice; *n* = 8 mice for AAV-D7-treated 28-mo mice). ∗p < 0.05, ∗∗p < 0.01, ∗∗∗p < 0.001 by one-way ANOVA followed by Student's t test post hoc analysis. N.S., not significant. See also Figure S1.

Here, we first examined whether therapeutic administration of AAV-D7 (*DOK7* gene therapy) enhances the activation of MuSK in muscle and the subsequent formation of NMJs in aged mice. Note that, although AAV9 vector targets several tissues, including skeletal muscle, heart, and liver in mice when delivered intravenously (Zincarelli et al., 2008), we previously reported that such systemic administration of AAV-D7 at postnatal day 5 caused no obvious abnormalities in motor function and histology of the major target tissues at 3 months of age (Arimura et al., 2014). Also, we reported that AAV-D7-treated *DOK7* myasthenia mice survived for at least 1 year with no apparent abnormality. AAV-D7 or control empty vector (AAV-ø) was intravenously administered to 24-mo male mice with a single dose of 4.8 × 10^13^ viral genomes per kilogram of body weight (vg/kg BW). Four months after the administration, we confirmed that, compared with non-treated 24-mo and AAV-ø-treated 28-mo mice, AAV-D7-treated 28-mo mice showed robust enhancement of MuSK activation, as judged by phosphorylation of MuSK and AChR in the hindlimb muscle (Figure 1A). Phosphorylation of the latter is known to be triggered by activation of MuSK (Fuhrer et al., 1997). To evaluate the effect of AAV-D7 treatment on the morphology of NMJs in aged mice, we stained frozen sections of the tibialis anterior (TA) muscle with α-bungarotoxin (α-Btx) to visualize AChR clusters (endplates) in myofibers, and with antibodies against synapsin-1 to label presynaptic motor nerve terminals (Figure 1B). Consistent with our previous reports on much younger mice (Arimura et al., 2014; Miyoshi et al., 2017), AAV-D7 treatment significantly increased the postsynaptic area characterized by clustered AChRs as well as the area of presynaptic, synapsin-1-positive motor nerve terminals at NMJs (Figures 1C and 1D), demonstrating that AAV-D7 treatment enlarges NMJs in aged mice. Furthermore, we found that AAV-D7 treatment did not significantly change the cover ratio of presynaptic motor nerve terminals to AChR clusters (Figure 1E). In addition, we also evaluated AChR cluster fragmentation, a structural hallmark of NMJs in aged rodents (Valdez et al., 2010), and found that AAV-D7 treatment did not significantly affect fragmentation of AChR clusters (Figure 1F). It is of note that a gradual increase in the percentage of denervated NMJs is also one of the typical alterations of NMJs with aging in mice, as it is in a mouse model of ALS (Valdez et al., 2010, 2012). Since AAV-D7 treatment not only enlarges NMJs but also suppresses NMJ denervation in ALS model mice (Miyoshi et al., 2017), we asked whether AAV-D7 treatment might also suppress NMJ denervation in aged mice. Indeed, the percentage of denervated NMJs in AAV-ø-treated 28-mo mice increased 4 months after the administration of AAV, whereas AAV-D7-treated 28-mo mice showed greatly reduced NMJ denervation compared with AAV-ø-treated 28-mo mice (Figure 1G). Moreover, AAV-D7-treated mice showed significantly lower denervation than non-treated 24-mo mice, suggesting that AAV-D7 treatment promotes reinnervation of denervated myofibers. In addition to the fast-twitch TA muscle, we assessed the effect of AAV-D7 treatment on the morphology of NMJs in the slow-twitch soleus muscle and found that AAV-D7 treatment also increased the areas of presynaptic motor nerve terminals and AChR clusters and reduced the percentage of denervated NMJs, without significantly changing the cover ratio and fragmentation levels of AChR clusters in soleus muscle (Figure S1), demonstrating similar effects of AAV-D7 treatment on fast- and slow-twitch muscles. Together, these findings indicate that *DOK7* gene therapy activates MuSK, enlarges NMJs, and enhances NMJ innervation in aged mice.

### *DOK7* Gene Therapy Enhances Compound Muscle Action Potential Amplitudes in Aged Mice

**Figure 2:**
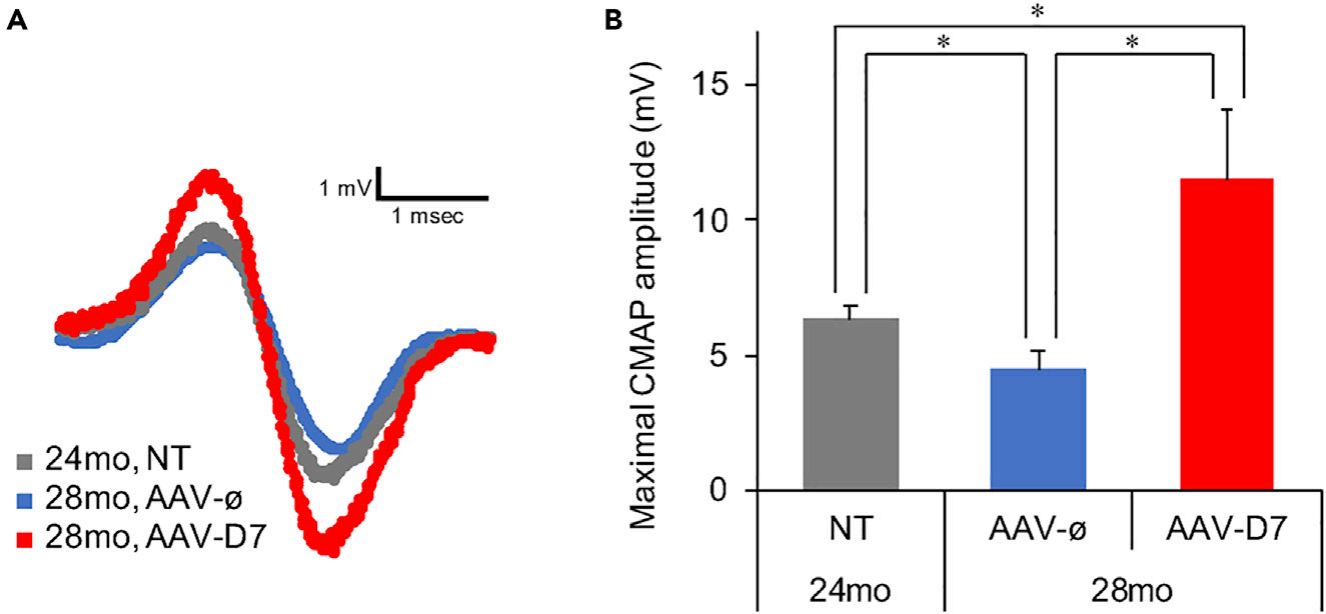
AAV-D7 Treatment Enhances the Maximal Amplitudes of CMAPs in Aged Mice Twenty-four month-old male mice were treated with AAV-D7 or the control empty vector (AAV-ø), and the maximal amplitudes of compound muscle action potentials (CMAPs) of TA muscles at 10 Hz stimulations were measured at 24 (NT, non-treated) or 28 months of age (AAV-D7- or AAV-ø-treated). Representative traces (A) and quantitative data (B) are shown. Error bars indicate means ± SEM (*n* = 15 mice for NT 24-mo mice; *n* = 10 mice for AAV-ø-treated 28-mo mice; *n* = 14 mice for AAV-D7-treated 28-mo mice). ∗p < 0.05 by one-way ANOVA followed by Student's t test post hoc analysis.

Motor neurons, when electrically excited by a nerve impulse, release the neurotransmitter acetylcholine to activate AChRs clustered on the postsynaptic membrane at the NMJ, leading to the generation of compound muscle action potentials (CMAPs), the amplitude of which depends on the number of myofibers firing action potentials (Willadt et al., 2018). Of note, several studies have reported that the maximal amplitude of CMAPs decreases with aging in mammals (Kurokawa et al., 1999; Pannérec et al., 2016), consistent with the increased percentage of denervated NMJs (Wu et al., 1996). Since NMJ innervation in aged mice was enhanced by AAV-D7 treatment (Figures 1G and S1F), we hypothesized that neuromuscular transmission and subsequent firing of muscle action potentials would also be enhanced in AAV-D7-treated 28-mo mice. Thus, we tested whether the maximal amplitude of CMAPs increases upon the AAV-D7 treatment in aged mice. The CMAPs were obtained with recording electrodes inserted into the TA muscle upon sciatic nerve stimulation. The maximal amplitude of CMAPs in control AAV-ø-treated 28-mo mice was lower than that in non-treated 24-mo mice, whereas AAV-D7-treated 28-mo mice displayed a higher amplitude compared with non-treated 24-mo and AAV-ø-treated 28-mo mice (Figure 2). Together with the enhanced NMJ innervation in AAV-D7-treated mice (Figures 1G and S1F), these results indicate that AAV-D7 treatment enhances muscle action potentials evoked by nerve stimulation in aged mice, most likely due to enhanced neuromuscular transmission.

### *DOK7* Gene Therapy Enhances Motor Function and Muscle Strength in Aged Mice

**Figure 3:**
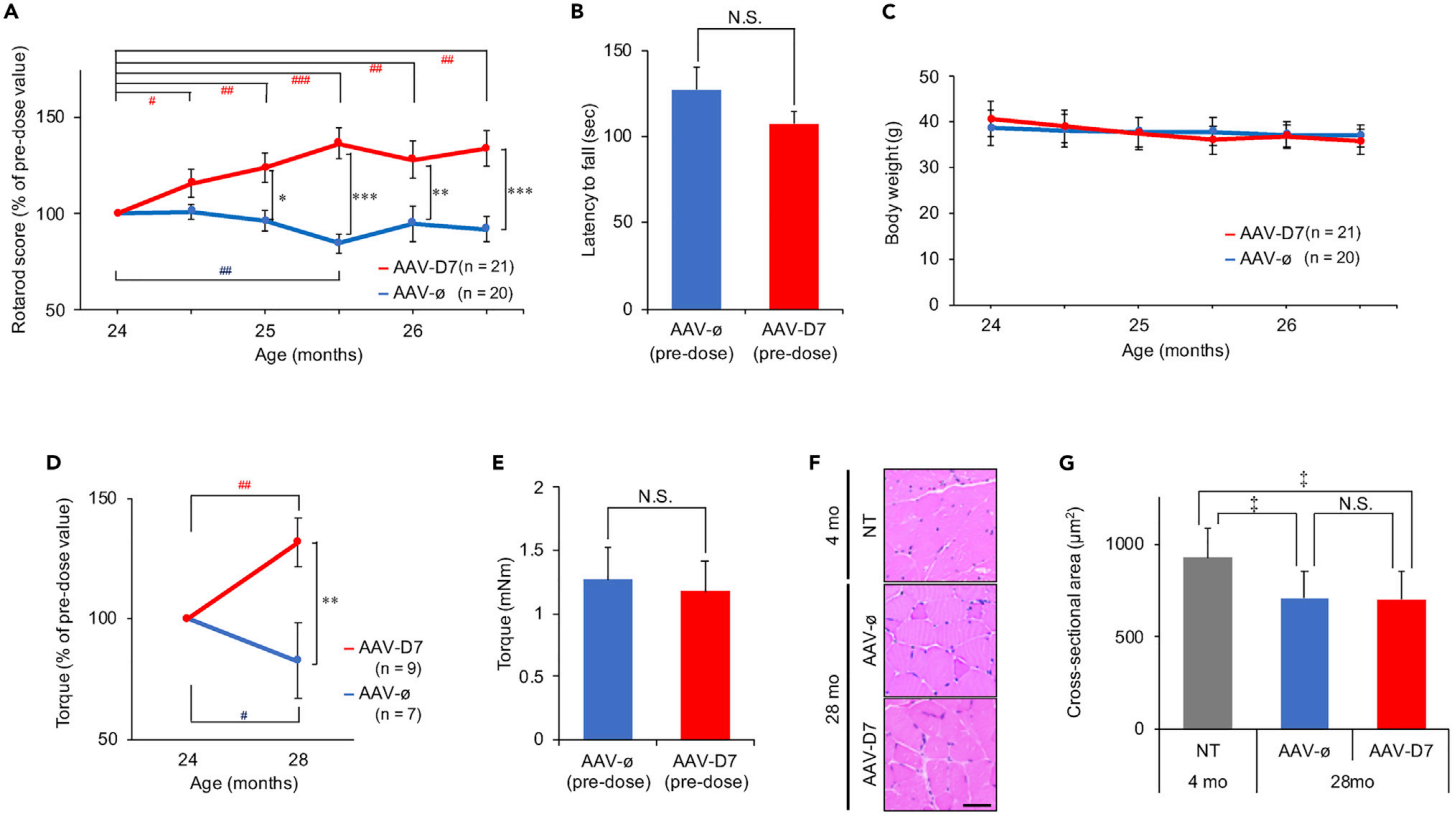
AAV-D7 Treatment Enhances Motor Function and Muscle Strength in Aged Mice Twenty-four month-old male mice were treated with AAV-D7 or AAV-ø and subjected to the following assays at the indicated ages. (A and B) Motor performance of pre-dose aged mice or those treated with AAV-D7 or AAV-ø were evaluated by rotarod test at the indicated ages (pre-dose mice, 24 months of age; treated mice, 24.5–26.5 months of age). Each score is expressed as relative value (%) of the latency to fall off the rotarod to pre-dose value of the individual mice at 24 months of age (A), and the absolute values of pre-dose mice are shown (B). Error bars indicate means ± SEM (*n* = 20 mice for AAV-ø-treated mice; *n* = 21 mice for AAV-D7-treated mice). (C) Body weight of pre-dose aged mice or those treated with AAV-D7 or AAV-ø at the indicated ages (pre-dose mice, 24 months of age; treated mice, 24.5–26.5 months of age) was measured. Error bars indicate means ± SEM (*n* = 20 mice for AAV-ø-treated mice; *n* = 21 mice for AAV-D7-treated mice). There is no statistically significant difference between the two tested groups at each time point (Student's t test). (D and E) Maximal isometric torque of pre-dose aged mice at 24 months of age or those treated with AAV-D7 or AAV-ø at 28 months of age (4 months after the administration of AAV) was measured. Each score is expressed as relative value (%) of the plantarflexion torque to pre-dose value of the individual mice at 24 months of age (D), and the absolute values of pre-dose mice are shown (E). Error bars indicate mean ± SEM (*n* = 7 mice for AAV-ø-treated mice; *n* = 9 mice for AAV-D7-treated mice). (F and G) Transverse sections of TA muscle of non-treated (NT) 4-mo mice and 28-mo mice treated with AAV-D7 or AAV-ø were stained with hematoxylin and eosin (F), and the myofiber cross-sectional area (CSA) was quantified (G). Scale bar, 100 μm. Error bars indicate means ± SEM (*n* = 10 mice for NT 4-mo mice; *n* = 8 mice for AAV-ø-treated 28-mo mice; *n* = 8 mice for AAV-D7-treated 28-mo mice). ∗p < 0.05, ∗∗p < 0.01, ∗∗∗p < 0.001 by Student's t test. ^#^p < 0.05, ^##^p < 0.01, ^###^p < 0.001 by paired Student's t test. ^‡^p < 0.05 by one-way ANOVA followed by Student's t test post hoc analysis. N.S., not significant.

To determine whether AAV-D7 treatment improves motor function in aged mice, we compared the motor performance of AAV-D7-treated mice with that of AAV-ø-treated mice using the rotarod test, in which the latency of mice to fall off the rod rotating under continuous acceleration was measured. Figure 3A shows that AAV-D7-treated mice significantly outperformed AAV-ø-treated mice in motor performance at each time point from 25 to 26.5 months of age (from 1 to 2.5 months after the administration of AAV), whereas no significant difference was observed between the two tested groups before AAV-ø or AAV-D7 treatment at 24 months of age (Figure 3B). Furthermore, although the rotarod test score in AAV-ø-treated mice was similar to or lower than that before treatment (at 24 months of age) at each time point throughout the test period, AAV-D7-treated mice showed a significant increase in their motor performance even at 24.5 months of age, or 0.5 months after treatment, in comparison with that before treatment. Given that no significant difference was observed in body weight between the AAV-ø- and AAV-D7-treatment groups throughout the test period (Figure 3C), these results together indicate that *DOK7* gene therapy enhances motor function in aged mice.

However, given that the motor performance determined by rotarod test (the ability of mice to maintain their balance on the rotating rod) is affected by many factors including altered cerebellar or spinal cord function (Günther et al., 2012; Sadakata et al., 2007), we cannot completely exclude the possibility that *DOK7* gene therapy improved motor performance in aged mice via altered function of non-skeletal muscle tissues. Thus, to directly test muscle strength, we measured the *in vivo* twitch force of hindlimb muscle upon electrical stimulation. By directly stimulating gastrocnemius muscle using electrodes in contact with skin over the muscle, we measured the force of plantarflexor muscles with minimal invasiveness before and after administration of AAV-D7 or AAV-ø. As evident in Figure 3D, AAV-D7-treated 28-mo mice showed significantly higher muscle strength than AAV-ø-treated 28-mo mice, whereas no significant difference was observed between pre-dose twitch forces of the two tested groups obtained before AAV-ø or AAV-D7 treatment at 24 months of age (Figure 3E). Furthermore, the muscle strength in AAV-ø- or AAV-D7-treated 28-mo mice was reduced to 83% or increased to 132%, significantly and respectively, as compared with each strength before treatment (at 24 months of age) (Figure 3D). Together these results indicate that *DOK7* gene therapy enhances both motor function and muscle strength in aged mice.

Consistent with numerous studies demonstrating that denervation induces not only loss of NMJs but also muscle atrophy (Guth et al., 1964), AAV-D7 treatment suppressed NMJ denervation and myofiber atrophy in a mouse model of ALS (Miyoshi et al., 2017). Thus, we thought that *DOK7* gene therapy would suppress the loss of muscle mass in aged mice and examined transverse sections of TA muscle in non-treated 4-mo and AAV-ø- or AAV-D7-treated 28-mo mice (Figure 3F). As expected, the myofiber cross-sectional area (CSA) was significantly reduced in AAV-ø-treated 28-mo mice compared with that in non-treated 4-mo mice (Figure 3G). However, the CSA in AAV-D7-treated 28-mo mice was similar to that in AAV-ø-treated 28-mo mice (Figure 3G), indicating that the enhancement of motor function and muscle strength does not need detectable muscle hypertrophy. Given that muscle strength is affected not only by muscle mass but also by its mechanical property (González et al., 2000), AAV-D7 treatment may enhance muscle strength by altering an as yet unidentified mechanical property of myofibers in aged mice (see below).

## Discussion

A growing body of evidence indicates that rodent NMJs undergo age-related structural alterations including fragmentation of AChR clusters and NMJ denervation (Valdez et al., 2010). Although the fragmentation of AChR clusters per se is not directly associated with the impairment of neuromuscular transmission (Willadt et al., 2016, 2018), NMJ denervation inevitably leads to the loss of NMJ functions and muscle weakness, highlighting the NMJ as a potential therapeutic target for age-related motor dysfunction (Taetzsch and Valdez, 2018). However, in contrast to intensive studies performed on the effects of aging on rodent NMJs, only a limited number of studies are reported on human NMJs. Two studies on postmortem human muscle tissues revealed age-related fragmentation of endplates, which is common in aged rodents (Oda, 1984; Wokke et al., 1990). Contradicting this, another group studied the morphology of human NMJs using lower limb muscle tissues after amputation surgery and reported that human NMJs throughout the adult lifespan (mean age, 67 years old; range, 34–92 years old) remain devoid of any of the age-related changes studied such as fragmentation of endplates, although denervation rate per se was not investigated (Jones et al., 2017). However, it might be better to interpret the data with some caution, because (1) the analyzed tissues were from patients with peripheral vascular disease or diabetes mellitus and thus probably affected by their pathological conditions, and (2) the diameter of myofibers or of motor axons in older age groups was not decreased, whereas it is established that myofibers and motor axons degenerate as humans advance into old age (Azzabou et al., 2015; Cartee et al., 2016; Galbán et al., 2007). Furthermore, a recent study suggests that failure to reinnervate denervated myofibers leads to reduced muscle strength in patients with sarcopenia (Piasecki et al., 2018). Consistent with this, in rodents, it has been established that progressive NMJ denervation and reduction of muscle strength occur with aging (Chai et al., 2011; Valdez et al., 2010). These observations suggest that age-related motor impairments are caused, at least in part, by NMJ denervation in mammals, although further investigations are required to fully understand age-related alterations of human NMJs.

As mentioned above, we previously demonstrated that AAV-D7 treatment enlarges NMJs, ameliorates the shortened life span, and improves the impaired motor activity in mouse models of neuromuscular disorders with NMJ defects, such as *DOK7* myasthenia, autosomal dominant Emery-Dreifuss muscular dystrophy, and ALS (Arimura et al., 2014; Miyoshi et al., 2017). In addition, other groups have shown that treatment with an agonist antibody to MuSK preserves NMJ innervation in a mouse model of ALS, although its effects on neuromuscular transmission and survival are controversial (Cantor et al., 2018; Sengupta-Ghosh et al., 2019). Furthermore, administration of a stabilized form (NT-1654) of the C-terminal 44 kDa fragment of motor neuron-derived agrin, a MuSK activator, enhances NMJ formation and improves motor behavior and survival of a mouse model of spinal muscular atrophy (Boido et al., 2018). These data together demonstrate that the NMJ is a promising therapeutic target in an array of neuromuscular diseases with NMJ defects. However, the effect of *DOK7* gene therapy on age-related motor dysfunction was unknown. In the present study, we demonstrated that a single-dose systemic administration of AAV-D7 to 24-mo mice enhanced MuSK activation and NMJ innervation in the aged mice (Figures 1A, 1B, 1G, S1A, and S1F) and augmented motor function and muscle strength (Figures 3A and 3D). Therefore, *DOK7* gene therapy exerts therapeutic effects on the muscle weakness and motor dysfunction of aged mice, although the mechanisms by which AAV-D7 treatment enhances NMJ innervation remain to be elucidated. We have previously shown that muscle-specific overexpression of Dok-7 induces the enlargement of not only the postsynaptic area but also the presynaptic motor nerve terminal (Inoue et al., 2009; Tezuka et al., 2014), suggesting that Dok-7 expression in muscle activates retrograde signaling from muscle to motor neurons. This retrograde signaling may contribute to NMJ innervation in aged mice by suppressing the degeneration of presynaptic motor nerve terminals and/or by accelerating the reinnervation of denervated myofiber. Interestingly, mice overexpressing Dok-7 specifically in muscle or those treated systemically with AAV9 expressing Dok-7-EGFP showed normal motor activity at 3 months of age (Arimura et al., 2014), when little or no denervation of NMJs is observed in wild-type mice.

The formation and maintenance of NMJs require not only Dok-7 but also Lrp4, a receptor of agrin for MuSK activation and also an important retrograde signal to induce presynaptic specialization of motor nerve terminals at NMJs (Wu et al., 2012; Yumoto et al., 2012). A paper recently reported reduced levels of Lrp4 protein expression and MuSK activation in muscle of aged mice, suggesting impaired MuSK-mediated signaling due to loss of Lrp4 protein. Indeed, the authors demonstrated that transgenic expression of Lrp4 in skeletal muscle from the embryonic stage alleviates NMJ denervation and improves neuromuscular transmission and muscle strength in aged mice (Zhao et al., 2018). Also, they found that Lrp4 interacts with sarcoglycan α (SGα) and demonstrated that intramuscular injection with an AAV serotype 9 vector expressing SGα fused with green fluorescent protein (GFP) (AAV9-SGα-GFP) at 22.5 months of age resulted in increased NMJ innervation, neuromuscular transmission, and muscle strength as compared with the AAV9-GFP-treated controls in 24-mo mice. However, they did not examine any enhancement of these values in comparison with each pre-dose value. Also, they examined neither MuSK activation nor motor function upon AAV9-SGα-GFP treatment or transgenic expression of Lrp4, unlike the present study on *DOK7* gene therapy (Figures 1A and 3A). Interestingly, the transgenic expression of Lrp4 in muscle or administration of AAV9-SGα-GFP induced a significant increase in the CSA of the TA muscle in aged mice, in contrast to *DOK7* gene therapy (Figures 3F and 3G), suggesting distinct mechanisms by which AAV-D7 administration enhances muscle strength. As mentioned, the decrease in muscle strength with aging is known to be attributed not only to the decrease in muscle mass but also to altered mechanical properties of the myofiber; the force generated by myofibers normalized to their CSA (specific force) declines with aging (Bruce et al., 1989; González et al., 2000; Morse et al., 2005; Urbanchek et al., 2001). Given that AAV-D7 treatment enhanced muscle strength in response to direct muscle stimulation (Figure 3D), *DOK7* gene therapy likely increases the specific force per se by improving such mechanical properties of myofibers, which could be due to fiber type alteration of the muscle*.* In addition, because AAV-D7-treated aged mice showed increases in NMJ innervation and CMAP amplitude (Figures 1B, 1G, 2A, 2B, S1A, and S1F), this treatment likely increases the number of functional NMJs, leading to enhanced muscle strength. Furthermore, because overexpression of Dok-7 in skeletal muscle enhances neuromuscular transmission at individual NMJs (Eguchi et al., 2020), AAV-D7 treatment may also strengthen neuromuscular transmission at individual NMJs in aged mice. The precise mechanisms underlying AAV-D7-mediated enhancement of muscle strength without significant muscle hypertrophy in aged mice awaits further studies.

### Limitations of the Study

Our findings established proof of principle that *DOK7* gene therapy, or potentially other methods that are able to enhance NMJ innervation, may be a novel treatment approach for age-related motor dysfunction. However, *DOK7* gene therapy has limitations in precise control of the duration and level of therapeutic intervention. Although this gene therapy benefited *DOK7* myasthenia or aged mice for at least 12 or 4 months, respectively, with no apparent abnormality (Arimura et al., 2014; the present study), there remains a potential risk of sustained adverse side effects, such as genotoxicity, in aged humans (Wang et al., 2019). Thus, it would be important to develop compound-based treatment in parallel with gene therapy. However, because age-related motor dysfunction is a progressive, multifactorial disorder, NMJ-targeting therapy per se can serve only as a symptomatic treatment for the age-related dysfunction, but not as a cure. Indeed, age-related loss of skeletal muscle mass and function is associated with many factors including reduced synthesis of myofibrillar proteins, mitochondrial dysfunction, decreased contractile properties, a shift to slower fiber types, and abnormal energy metabolism (Larsson et al., 2019). Given that several groups have developed treatments aimed at inducing muscle hypertrophy for age-related loss of skeletal muscle mass and subsequent motor dysfunction (Fujii et al., 2017; Vinel et al., 2018), *DOK7* gene therapy or other NMJ-targeted therapies might be more effective for age-related motor dysfunction when used in combination with other non-NMJ-targeting therapeutics, such as those aimed at inducing muscle hypertrophy.

### Resource Availability

#### Lead Contact

Further information and requests for resources and reagents should be directed to and will be fulfilled by the Lead Contact, Yuji Yamanashi (yyamanas@ims.u-tokyo.ac.jp).

#### Materials Availability

All unique/stable reagents generated in this study are available from the Lead Contact with a completed Materials Transfer Agreement.

#### Data and Code Availability

There is no dataset and/or code associated with the article.

## Methods

All methods can be found in the accompanying Transparent Methods supplemental file.

## Supplemental Information

Document S1. Transparent Methods and Figure S1
