# Supplementary material for: DOK7 Gene Therapy Enhances Neuromuscular Junction Innervation and Motor Function in Aged Mice
Source: iScience. 2020 Aug 5;23(8):101385. doi: 10.1016/j.isci.2020.101385 (PMC7452162; doi:10.1016/j.isci.2020.101385)
Supplement: Document S1. Transparent Methods and Figure S1 [file mmc1.pdf]

iScience, Volume *23*

Supplemental Information

DOK7 Gene Therapy Enhances Neuromuscular Junction Innervation and Motor Function in Aged Mice

Ryo Ueta, Satoshi Sugita, Yoshihiko Minegishi, Akira Shimotoyodome, Noriyasu Ota, Noboru Ogiso, Takahiro Eguchi, and Yuji Yamanashi

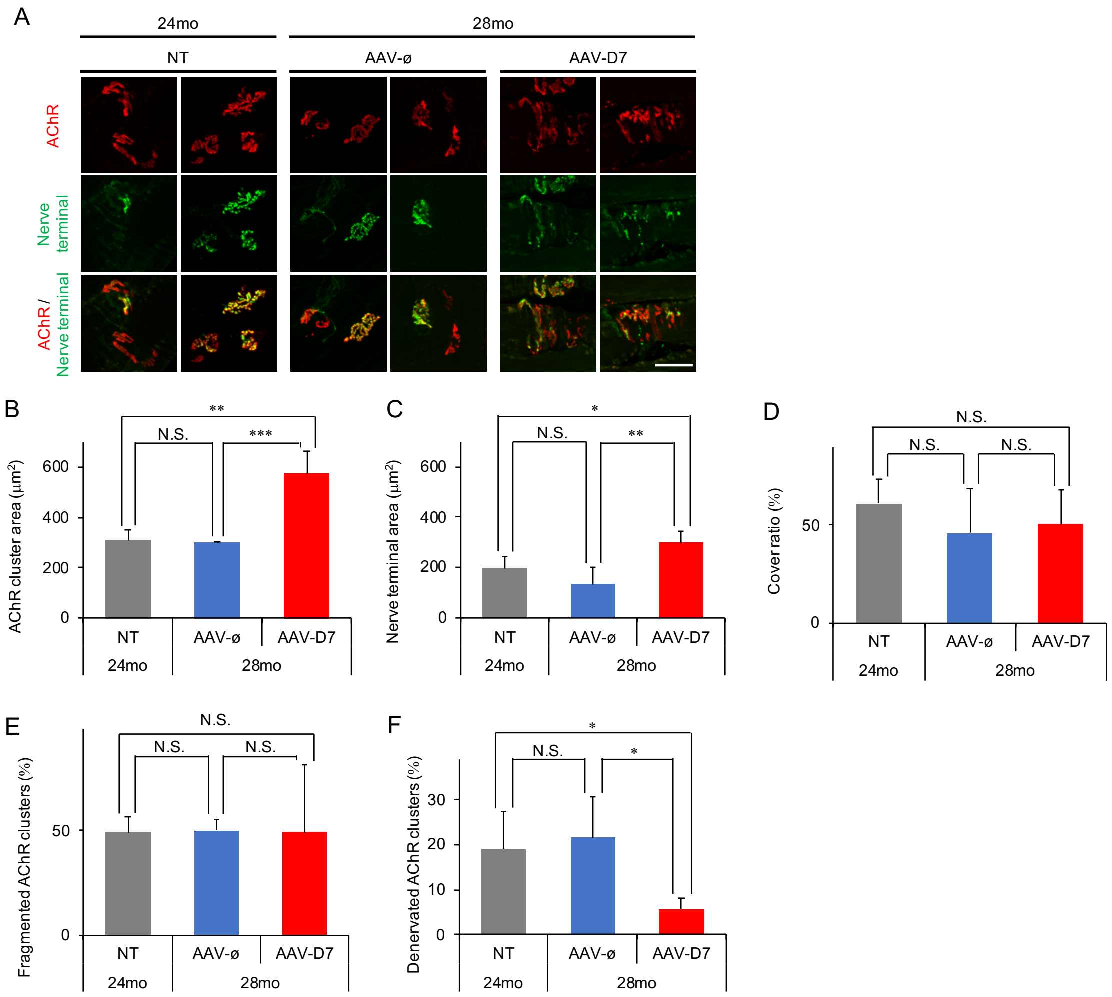

Figure S1. AAV-D7 treatment enhances NMJ innervation in soleus muscle of aged mice, Related to Figure 1.

Twenty-four month-old (mo) male mice were treated with AAV-D7 or the control empty vector (AAV-ø), and subjected to the following assays at 28 months of age (4 months after the administration of AAV).

(A-F) Longitudinal sections of soleus muscles of non-treated (NT) 24 mo mice or 28 mo mice treated with AAV-D7 or AAV-ø were stained as in Figure 1B, and representative images are shown (A) (Scale bar, 50 μm). The area of AChR clusters (B) and motor nerve terminals (C), the cover ratio (D), and the percentage of fragmented AChR clusters (E) and denervated AChR clusters (F) were quantified. Error bars indicate means ± SEM (n = 4 mice for NT 24 mo mice; n = 4 mice for AAV-ø-treated 28 mo mice; n = 4 mice for AAV-D7-treated 28 mo mice). *P < 0.05, **P < 0.01, ***P < 0.001 by one-way ANOVA followed by Student's t-test post-hoc analysis. N.S., not significant.

Transparent Methods

Mice

Male C57BL6/N were purchased from Japan SLC and maintained in the Experimental Animal Facility of the National Center for Geriatrics and Gerontology (NCGG) until experimental use. Mice were housed on a 12/12-hour light/dark cycle in specific pathogen-free conditions with free access to water and standard mouse chows. All animal experiments were conducted in the Laboratory Animal Research Center of The Institute of Medical Science, The University of Tokyo, in the Experimental Animal Facility of NCGG, or in the Experimental Animal Facility of Kao Corporation's R&D Department. All animal studies were performed in accordance with the guidelines for animal care and use of each institute, and approved by the institutional animal care and use committees.

AAV production and injection

The cDNA encoding human Dok-7 cDNA tagged with c-myc epitope was cloned into pAAV-MCS (Agilent Technologies), which carries the cytomegalovirus promoter, to obtain pAAV-Dok-7-myc plasmid. For production of AAV-D7, HEK293EB cells were co-transfected with the AAV9 chimeric helper plasmid pRep2Cap9, the adenovirus helper plasmid pHelper (Agilent Technologies), and pAAV-MCS or pAAV-Dok-7-myc in a HYPERFlask vessel (Corning) using polyethylenimine, and cultured for 5 days (Lin et al., 2007; Matsushita et al., 2004). The AAV particles were purified by density-gradient ultracentrifugation (Tomono et al., 2016). The viral titers were determined by real-time quantitative PCR using AAVpro Titration Kit (Takara Bio). 4.8×10^{13} vg/kg body weight of AAV-D7 or AAV-ø were intravenously injected by a single dose via the tail vein.

Immunoprecipitation and Western blotting

Tissue lysates were prepared from hindlimb muscle with alkaline lysis buffer [50 mM Tris·HCl (pH 9.5), 1% sodium deoxycholate, Complete protease inhibitor (Roche), PhosSTOP phosphatase inhibitor (Roche)]. For immunoprecipitation, lysates were incubated with antibodies to MuSK (N-19 and C-19) (Santa Cruz Biotechnology) or AChRβ1 (H-101) (Santa Cruz Biotechnology), followed by incubation with protein G-Sepharose (GE Healthcare). The immune complexes were washed five times and collected as immunoprecipitates. For Western blotting, immunoprecipitates or lysates were separated

by SDS-PAGE on 6 or 9% gels and transferred to a PVDF membrane (Merck Millipore), which was then incubated with antibodies to phosphotyrosine (4G10) (Merck Millipore), MuSK (AF562) (R&D Systems), AChRβ1 (H-101) (Santa Cruz Biotechnology), Dok-7 (A-7) (Santa Cruz Biotechnology), or actin (I-19) (Santa Cruz Biotechnology), washed, and incubated with horseradish peroxidase-labeled anti-mouse (GE Healthcare) or anti-goat (Santa Cruz Biotechnology) IgG. The blots were visualized using a LAS4000 imager with ECL Prime Western Blotting Detection Reagent (GE Healthcare).

Immunohistochemistry of NMJs

Mice were anesthetized and perfused through the heart with PBS. Skeletal muscles were dissected out, embedded in Tissue-Tek OCT compound (Sakura Finetek) and processed for cryostat sectioning. 30-μm longitudinal cryosections were blocked in PBS containing 2% BSA and 0.1% Triton X-100. No fixation was performed at any point in the process. Sections were sequentially incubated with primary antibodies overnight at 4 ºC, washed with PBS, and incubated in a mixture of Alexa Fluor 488-conjugated secondary antibodies (Thermo Fischer Scientific) and CF 594-conjugated α-Bungarotoxin (Biotium) overnight at 4 ºC. After washing, the sections were mounted with Vectashield (Vector Laboratories). Confocal Z serial images were collected with an FV1000 Confocal Laser Scanning Microscope (Olympus) and collapsed into a single image. Images were captured with the same settings and exposure time in each experimental group for comparison. The sizes (areas) of presynaptic motor nerve terminals and postsynaptic AChR clusters, and the cover ratio of presynaptic motor nerve terminals to AChR clusters were quantified using cellSens Digital Imaging Software (Olympus). For quantification, seven to thirty microscopic fields with the 20× objective were chosen at random on the tibialis anterior and soleus muscle from each mouse, and more than 100 synaptic sites were analyzed per muscle for each mouse. The AChR cluster was scored as fragmented when it consisted of five or more segments, and the NMJ was scored as denervated when no presynaptic, synapsin-1-positive area was detected on the AChR cluster area. These experiments were conducted in a blinded fashion.

Electromyography

Compound muscle action potentials (CMAPs) were studied using a PowerLab 26T data acquisition system (ADInstruments). Mice were anesthetized using isoflurane inhalation,

and the sciatic nerve was exposed at the left mid-thigh. Paired stimulating electrodes separated by 3 mm were kept in contact with the exposed sciatic nerve at 10 mm from the midline for supramaximal stimulation at 10 Hz. The recording electrodes were inserted in the middle of the left tibialis anterior muscle whereas the reference one was inserted 5-mm distally, both of which were connected via an MPA8I preamplifier (Multi Channel Systems) to an SC8x8BC signal collector (Multi Channel Systems). To isolate stimulus artifacts, a ground electrode was placed between the stimulus and recording electrodes. CMAPs were recorded for 1 second, and peak–peak amplitudes were determined in LabChart software (ADInstruments). The CMAP amplitude for the first stimulation was analyzed. These experiments were conducted in a blinded fashion.

Rotarod test

Mice were placed on a rotating cylinder (MK-610A) (Muromachi Kikai), and the latency to fall was recorded. The device was set to accelerate from 4 to 40 rpm over a 5-min period. Before testing, each mouse was acclimated to the rotarod device for three trials per day on three consecutive days to familiarize the mice with the device and test protocols. The test was performed immediately before administration of the AAV and every 0.5 months thereafter until 2.5 months after the administration. The measurement was performed three times each day, and the average of the individual mice's measured values was calculated and estimated as the index of motor performance. These experiments were conducted in a blinded fashion.

Measurement of maximal plantarflexion isometric torque

Maximal plantarflexion isometric torque was measured with a slight modification of the method previously described (Itoh et al., 2017). Briefly, under anesthesia with isoflurane, electrical stimulation was applied to the posterior surface of the skin of the lower limbs. To attach surface stimulation electrodes (Bio Research Center) to the skin, viscous electrical conductive gel (CR) (Sekisui Plastics) was applied between the electrodes and the skin. The electrodes were fixed with adhesive tape to the surface of the myotendinous junction and a 5-mm proximal locus. Plantarflexor muscles were percutaneously stimulated via surface stimulation electrodes, and maximal plantarflexion was evoked using a supramaximal twitch current (100-Hz frequency, 1.0-msec duration, and 10.0-mA current). Isometric plantarflexion torque (T) was calculated from the pressure applied to a footplate (F) and the

distance from the axis of the ankle joint to the sensor (r) as follows: $T = Fr$. These experiments were conducted in a blinded fashion.

Supplemental References

Itoh, Y., Murakami, T., Mori, T., Agata, N., Kimura, N., Inoue-Miyazu, M., Hayakawa, K., Hirano, T., Sokabe, M., and Kawakami, K. (2017). Training at non-damaging intensities facilitates recovery from muscle atrophy. Muscle Nerve 55, 243–253.

Lin, J., Zhi, Y., Mays, L., and Wilson, J.M. (2007). Vaccines Based on Novel Adeno-Associated Virus Vectors Elicit Aberrant $CD8^+$ T-Cell Responses in Mice. J. Virol. 81, 11840–11849.

Matsushita, T., Okada, T., Inaba, T., Mizukami, H., Ozawa, K., and Colosi, P. (2004). The adenovirus E1A and E1B19K genes provide a helper function for transfection-based adeno-associated virus vector production. J. Gen. Virol. 85, 2209–2214.

Tomono, T., Hirai, Y., Okada, H., Adachi, K., Ishii, A., Shimada, T., Onodera, M., Tamaoka, A., and Okada, T. (2016). Ultracentrifugation-free chromatography-mediated large-scale purification of recombinant adeno-associated virus serotype 1 (rAAV1). Mol. Ther. Methods Clin. Dev. 3, 15058.
